# Manipulating Individual Decisions and Environmental Conditions Reveal Individual Quality in Decision-Making and Non-Lethal Costs of Predation Risk

**DOI:** 10.1371/journal.pone.0052226

**Published:** 2012-12-13

**Authors:** Robert L. Thomson, Gustavo Tomás, Jukka T. Forsman, Mikko Mönkkönen

**Affiliations:** 1 Section of Ecology, Department of Biology, University of Turku, Turku, Finland; 2 Department of Evolutionary Ecology, Museo Nacional de Ciencias Naturales, Madrid, Spain; 3 Department of Biology, University of Oulu, Oulu, Finland; 4 Department of Biological and Environmental Science, University of Jyväskylä, Jyväskylä, Finland; Liverpool John Moores University, United Kingdom

## Abstract

Habitat selection is a crucial decision for any organism. Selecting a high quality site will positively impact survival and reproductive output. Predation risk is an important component of habitat quality that is known to impact reproductive success and individual condition. However, separating the breeding consequences of decision-making of wild animals from individual quality is difficult. Individuals face reproductive decisions that often vary with quality such that low quality individuals invest less. This reduced reproductive performance could appear a cost of increased risk but may simply reflect lower quality. Thus, teasing apart the effects of individual quality and the effect of predation risk is vital to understand the physiological and reproductive costs of predation risk alone on breeding animals. In this study we alter the actual territory location decisions of pied flycatchers by moving active nests relative to breeding sparrowhawks, the main predators of adult flycatchers. We experimentally measure the non-lethal effects of predation on adults and offspring while controlling for effects of parental quality, individual territory choice and initiation of breeding. We found that chicks from high predation risk nests (<50 m of hawk) were significantly smaller than chicks from low risk nests (>200 m from hawk). However, in contrast to correlative results, females in manipulated high risk nests did not suffer decreased body condition or increased stress response (HSP60 and HSP70). Our results suggest that territory location decisions relative to breeding avian predators cause spatial gradients in individual quality. Small adjustments in territory location decisions have crucial consequences and our results confirm non-lethal costs of predation risk that were expressed in terms of smaller offspring produced. However, females did not show costs in physiological condition which suggests that part of the costs incurred by adults exposed to predation risk are quality determined.

## Introduction

Habitat selection is an important decision in the life of any organism. Individual survival and future reproductive output are largely dependent on the quality of the habitat in which an individual exists. Breeding habitat selection can be particularly important because choices strongly influence reproductive success and fitness [1]. Individual habitat selection is a flexible decision-making process. Individuals gather information, via personal experience and through the use of cues that decrease the unpredictability of the choice [2], [3], [4].

Predation risk is an important component of habitat quality. Predation risk can alter breeding habitat selection by individuals of many taxa [5]–[8], and thereby alter the spatial structure and diversity of communities [9], [10]. For the individual, a poor choice in breeding habitat relative to ambient predation risk will have negative consequences, either through decreased survival or subtly via non-lethal costs [11]. Reproductive investment may be altered in terms of offspring number and invested resources resulting in reduced reproductive success in terms of smaller and fewer offspring when predation risk is greater [12]–[14]. Increased risk from predator presence also can increase physiological costs through a wide range of physiological responses [15] that often include an increased stress response [16]–[20]; but see [21], [22]. Among them, evaluation of heat-shock proteins (HSP, also called stress proteins) has gained attention as a valuable tool in ecological and evolutionary research in last decades [23], [24], [25]. Stress proteins function as a major molecular barrier to alterations in cellular homeostasis, and respond to a wide array of stress agents [24]. In natural bird populations, evidence of stress protein induction exists under nestling competition [26], higher parental effort [27], and parasitism [28], [29]. Stress protein induction relative to risk of predation has only recently been investigated in different taxa, including insects [18], crustaceans [19], amphibians [22], mammals [17], and birds [20].

For territorial prey species, we expect prey to use a range of mechanisms to detect and avoid high risk sites in their breeding location decisions [13], [30]–[32]. However, individuals differ in risk and this variance could result from some individuals accepting higher risk when resource availability is capable of offsetting the cost of predation risk. In this case, we would not expect large costs of variation in risk. On the other hand, individuals may differ in risk because of individual quality, where low quality individuals are relegated to high risk sites by territorial interactions or due to poorer capability to perceive risk and make adaptive behavioral adjustments. Such possible covariances have important consequences for estimating the true physiological and reproductive costs of risk alone. Individuals are faced with reproductive decisions (number of young, effort in caring for the young, etc.) that often vary with quality such that low quality individuals typically invest less (fewer young of poorer quality, greater stress for the female) [33]. This reduced reproductive performance could appear as a cost of increased risk but may simply reflect lower quality. Thus, controlling for individual quality is critical to assessing the fitness consequences of decisions [34] and isolating the true costs of predation risk alone to breeding individuals. Field experiments using free-ranging animals that control for individual quality and the effects of individual decision-making in space are needed to quantify the effect of predation risk.

The nests of avian predators appear to anchor predation risk in natural landscapes [35], [36]. Prey may use cues to avoid or optimize proximity to breeding predators, possibly causing individuals of different quality to be non-randomly distributed even at short distances. We previously found that pied flycatchers (*Ficedula hypoleuca*) show fine-tuned territory location decisions relative to predator nests [35], [36]. Individuals preferentially settled at intermediate distances from sparrowhawk (*Accipiter nisus*) nests. Sparrowhawks initiate breeding before flycatchers arrive to their breeding grounds and are the main predator of adult passerines in northern breeding bird communities. Reproductive output and parental condition of pied flycatchers decreased by up to 42% with increasing proximity within 300 m of sparrowhawk nests [20], [36], and parental behaviors changed by as much as 54% within this distance [20], [37]. Other small bird species have shown similar trends [38]. These results suggest substantial non-lethal costs of living in close proximity to a predator and argue against resource abundance offsetting risk costs (alternative 2 above). However, these correlative results do not separate any potential covariance of quality from risk on observed differences in performance. Thus, controlling for quality is vital to gaining a proper understanding of the non-lethal effects of predation risk, and for understanding whether lower quality individuals use sites that are more exposed to risk.

We take a novel experimental field approach to tease apart individual quality and risk by controlling individual quality relative to the decision making process. Quality is defined here as the ability of the individual to cope with a specific environment or stressor with minimal cost. In this study we alter the actual territory location decisions of breeding pied flycatchers relative to breeding sparrowhawk by altering the position of the nest in a forest patch. We moved nest boxes containing breeding flycatchers to measure the non-lethal effects of predation on adults and offspring while controlling for the effect of parental quality, individual territory choice and initiation of breeding.

We focused on physiological and reproductive measures that were explained by distance to sparrowhawk nests in previous studies, namely size of offspring, maternal condition, and stress protein response [20], [36]. We test if nest site risk, independent of parental quality will (i) impact the size of chicks and (ii) impact measures of maternal condition and stress. If risk is difficult to assess at the time of territory choice and individual quality varies randomly with respect to distance to sparrowhawk nests, we expect similar results to our previous correlative studies, which included a strong linear response. In particular, we expect that flycatchers nests moved closer to hawk nests will have smaller and fewer chicks, and that adult females attending these nests will show increased mass loss and stress protein response. In contrast, if individual quality is higher for females that breed farther from sparrowhawk nests, we expect the treatment costs for these females to be much lower.

## Materials and Methods

### Ethics statement

Blood sampling and nest moving were performed under permit from the Centre for Economic Development, Transport and the Environment for North Ostrobothnia: PPO-2004-L-196–254 and PPO-2005-L-269–254. Bird ringing was performed under licence number 2836, issued to the lead author from the Finnish Museum of Natural History (the custodian of bird ringing in Finland).

### General methods

We located seven sparrowhawk nests in the forests near Oulu, northern Finland (65°N, 25°30′E) in summers 2004 and 2005. The vicinity of sparrowhawk nests consisted of mixed forests with varying proportions of Scots pine (*Pinus sylvestris*), Norway spruce (*Picea abies*) and birch (*Betula* spp.). Sparrowhawk prey mainly on small songbirds and arrive on their territories in April and initiate egg-laying by early May. Pied flycatchers begin arriving in the study area from middle May, by which time sparrowhawks have initiated incubation. We placed between 8 and 12 nest boxes for flycatchers at 120–130 m from each sparrowhawk nest (in a circle around each nest). Flycatchers were allowed to freely settle in the nest boxes, and all resulting nests were closely monitored to determine the date the first egg was laid.

Prior to the experimental treatment, we paired flycatcher nests in the same predator territory based on the date of first egg. This pairing was done to control for arrival dates. Within pairs we randomly assigned the territory location manipulation treatment of high and low predation risk. Additional boxes in territories with odd numbers of nests were randomised for treatment and included in analyses; this prevented a paired design analysis. High risk treatment nests were moved towards the predator nest, while low risk treatment nests were moved away from the predator.

Flycatcher nests were moved during incubation, which involved moving boxes a short distance, about 10 m daily, for 7 or 8 days. Nest boxes were moved carefully both with the female on the nest and with the female not present. Boxes were not moved on colder days to avoid exposing incubated eggs. To our knowledge, moving the nests of pied flycatchers has only been used in three previous studies [39]–[41]; however, we are the first to move nests during incubation. This was done in order to control initial parental clutch investment into the breeding attempt, which would have differed if nests were moved during nest building as in the previous studies. A movement of 70–80 m in the position of the nest would alter the foraging sites regularly used by parents and therefore the effective territory, as nearest neighbour distances are frequently 50 m in this species (personal observation).

Nest moving created high risk treatments, with nests located within 50 m, and low risk treatments with nests located over 200 m from hawk nests. In most cases the nests were in their final position before the clutch hatched, however, three nests were moved an additional time after hatching. Flycatchers fully tolerated the moving of their nests and all nests moved in this study hatched.

Prior to moving the nests, on the 4^th^ or 5^th^ day of the incubation period, we trapped flycatcher females on the nest (simply removing the incubating female). A blood sample was immediately taken from the brachial vein, which was collected using a capillary tube (max. 100 *µl*) and placed in an eppendorf tube. Time between start of handling and blood sampling were not measured, but was roughly the same for all individuals and occurred within two or three minutes. Trapping protocols do not incur biases associated with stress capture when HSPs are evaluated due to the relatively slow response of stress proteins [25], [42], though this may be problematic with other stress measures such as corticosterone which represents a fast stress response. After blood sampling, females were weighed to the nearest 0.1 g, wing measurements taken to the nearest 1 mm and ringed with a numbered metal ring. Females were released back onto the nest. Nest moving started two days after this procedure.

Hatching dates were checked through daily visits. At 12 days old, chick mass, tarsus and wing length were measured and used as an indication of nestling quality. Prior to processing chicks, we captured adult females using nest box traps. A blood sample and body mass was again taken from females.

### HSP estimation

Blood samples were placed in a cool box in the field until they could be later prepared in the laboratory. Samples remained in the cool box for periods less than eight hours. During this time period there are no significant changes in HSP60 or HSP70 blood protein levels [42]. HSP levels were determined from the blood cellular fraction by means of Western blot. Samples of soluble proteins (70 μg/well) were separated by SDS-PAGE; this amount of total protein is in the linear range of the antibody-antigen response for the species and antibodies studied. We used anti-HSP70 (clone BRM22, Sigma H-5147) diluted 1/5000 and anti-HSP60 (clone LK2, Sigma H-3524) diluted 1/1000 primary monoclonal antibodies. The peroxidase-conjugated secondary antibody was goat anti-mouse specific for the Fc region (Sigma A-0168) at 1/6000 dilution. Protein bands were quantified using 1D image analysis software. For details see [42], [43].

### Statistical analysis

We used generalized linear mixed models (GLMMs) to analyse the effect of moving treatments on measures of nest success and maternal condition. Flycatcher nest was the sampling unit for overall nest success and all maternal measures. In these models, predator territory was included as a random factor to account for unexplained differences between the territories. Treatment and year were included as fixed factors. To test treatment effects on overall reproductive success in terms of number of chicks produced, the number of chicks at 12 days was modelled with a Poisson distribution.

Maternal body mass and stress protein levels (HSP70 and HSP60) were modelled with a normal distribution. For each maternal variable three models were run, one accounting for initial measures taken during incubation (prior to box moving), and one accounting for final measures during nestling provisioning (after moving). We also modelled the across season difference (initial measure minus final measure) in these variables, to further control for between-individual differences. Models included a term of clutch or brood size to account for the variation on maternal measures caused by the number of offspring in the nest (clutch size during incubation, brood size during nestling phases). To control for individual size in the maternal body mass model, we included wing length as a covariate in the model, which was kept in the final model. Treatment and year were included as fixed factors, and “Blot” was included as a fixed factor for stress protein measures, which stems from the Western-blot technique, where blots may show variation; [29]). Including “Blot” in analyses controls for this variation.

In models analysing chick quality, each chick was the sampling unit. Nest box was nested within predator territory and entered as a random factor in models. This structure accounts for both the fact that individual nestlings are linked by their common nest and that nests within the same sparrowhawk territory are linked. To test treatment effects on the quality of chicks produced chick mass, wing and tarsus length were modelled with a normal distribution. Brood size (number of chicks in the nest) was included as a continuous variable in all cases to account for the trade-off between brood size and growth. Treatment and year were included as fixed factors.

In all models, year was removed if non-significant to simplify models further. Other terms are of known importance were retained in final models. Kenward-roger method was used as degrees of freedom in all cases. Random terms proved unimportant with covariance parameter estimates having standard errors larger than the estimate in almost all cases. All GLMMs were run using SAS 9.2.

## Results

A total of 44 pied flycatcher pairs nested around seven sparrowhawk nests. Of these, 22 nests were moved towards the predator nest (high risk treatment), and 22 nests were moved away (low risk treatment). Three nests (two high risk and one low risk) failed to produce fledglings. In addition, four nests (one high risk and three low risk) were found to have only a female provisioning young. These nests were removed from response analyses.

Our moving box manipulations controlled for individual quality. No differences were found in the laying date of the first egg; both treatment groups averaged 3 June (df = 42, t = 0.56, p = 0.58). There were no differences in clutch size either; both high risk and low risk nests averaged 6.3 eggs per clutch (Kruskal-Wallis: χ^2^ = 0.35, df = 1, p = 0.56).

The number of 12 day old chicks produced by nests was not explained by treatment (F_1,37_ = 0.13, p = 0.72; LSmeans: high risk 5.3±0.5 vs. low risk 5.6±0.6). However, aspects of chick size measured at 12 days old were explained by the moving box treatment (Table 1). Chicks raised in high risk nests had significantly shorter tarsi and wings than chicks raised in low risk nests, although chicks mass was not explained by treatment. As might be expected, the number of chicks in the nest (brood size) also explained significant variation in chick size, with brood size negatively related to chick size (Table 1).

**Table 1:** Results of linear mixed models examining variables of chick size in nests moved to high risk sites close to sparrowhawk nests and nests moved to lower risk sites away from sparrowhawk nests.

Variable	df	F	P	Least Square mean estimates	
	Wing length			High risk	Low risk
Treatment	1, 27.1	7.18	0.01	48.0±0.5	50.1±0.6
Brood size	1, 31.7	27.78	<0.001		
	Tarsus length				
Treatment	1, 18.6	12.53	0.002	16.93±0.07	17.29±0.07
Brood size	1, 25.7	12.53	0.02		
	Mass				
Treatment	1, 30.7	1.33	0.26	13.91±0.18	14.21±0.19
Brood size	1, 36.6	0.67	0.42		

Maternal measures of condition and stress during incubation, prior to moving, were equal between treatments (Incubation mass LSmeans: high risk 15.09±0.18 g vs. low risk 14.82±0.18, F = 1.39, p = 0.3; Incubation HSP70: high risk 7617±159 vs. low risk 7705±150, F = 0.26, p = 0.6). This again suggests that parental quality was adequately controlled.

Final measures of maternal condition and stress were also not explained by the moving treatment. Female body mass during provisioning was the same between treatments (Table 2). In addition, female mass change across season was not explained by treatment (high risk 2.6±0.14 vs. low risk 2.7±0.15, F = 0.09, p = 0.77). Levels of stress protein 70 were not affected by the moving treatment (Table 2), nor the change in stress protein 70 across the season (high risk 446±241 vs. low risk 380±220, F = 0.06, p = 0.8). Stress protein 70 levels were however explained by the number of chicks in the nest (brood size) and as expected also by variation of different blot runs (Table 2). Increased brood size resulted in higher stress protein levels. All similar models run with stress protein 60 revealed no significant effects of moving treatment.

**Table 2:** Results of linear mixed models examining variables of maternal condition in nests moved to high risk sites close to sparrowhawk nests and nests moved to lower risk sites away from sparrowhawk nests.

Variable	df	F	P	Least Square mean estimates	
	Female mass			High risk	Low risk
Treatment	1, 32	1.95	0.17	12.5±0.13	12.2±0.14
Wing length	1, 32	2.12	0.16		
Chick number	1, 32	3.94	0.06		
	Female HSP70 level				
Treatment	1, 22.2	0.02	0.89	6996±509	7018±508
Blot	4, 22.8	4.28	0.01		
Chick number	1, 22.3	7.07	0.01		

## Discussion

Making adaptive territory location decisions relative to an environmental stressor such as predation risk is a vital first step to ensure high reproductive output. Our study found experimental evidence that such fine-scaled decisions were made by settling pied flycatchers. We found negative effects on the quality of offspring, in terms of smaller tarsi, produced in nests under high predation risk. The nest environment was manipulated by moving nests about 150 m towards a breeding predator. This unique treatment controlled for parental quality in terms of adaptive decision making relative to predation risk. Surprisingly, females attending nests in close proximity to predator nests did not show altered condition in terms of mass changes or stress protein induction, even though they produced on average smaller young.

Our results confirm real non-lethal fitness costs of predation risk in birds [44]. Territory location decisions even within the forest patch scale altered perceived predation risk, which altered the costs to breeding flycatchers. Despite parental quality being controlled, smaller offspring were produced in nests close to breeding avian predators, which suggest predator proximity increased the perception of risk. These non-lethal effects will also entail a long term cost to the parents and their offspring, because tarsus length in pied flycatcher nestlings is linked to the probability of offspring becoming breeders. Longer is ‘better’, with reduced survival for offspring with shorter tarsi [45]. Direct or non-lethal costs to offspring may be incurred in territories with higher predation risk even within the short distances of adjacent territories [16], [36], [46]. It is likely that the perceived predation risk negatively influenced offspring provisioning via lowered parental foraging efficiency; decreasing offspring size. Overall, we suggest that failure to adequately control for individual quality could lead to an overestimation of non-lethal costs in certain systems.

We found that female flycatchers did not suffer measurable physiological costs in high predation risk treatments. Body condition and stress protein levels were the same for individuals in the two treatments. This is in contrast with our earlier correlative study, where individuals breeding closer to predator nests showed significantly reduced body condition and increased stress protein induction at the end of the breeding cycle; even though initial measures did not suggest differences [20]. Crucially, however, we did not experimentally control for individual quality in that study. In the current experimental study, flycatchers manipulated to high risk sites did however experience fewer days close to hawk nests than flycatchers nesting at similar distances in the correlative study, but it is unlikely to explain the difference in results as no trends were even found in the measures tested.

Our results have two important implications. First, our experiment suggests that part of the cost incurred by adults exposed to predation risk is quality determined. This implies that there indeed are quality differences among individuals that are manifested in their capacity to make good habitat selection decisions. Individual quality stemming from differing territory location decisions appears to also be a relevant measure of individual ability to tolerate environmental stressors (predation risk), and maybe even balance better between current and residual reproductive value. In natural conditions, flycatchers choosing territories and breeding in sites that optimize predation risk generally produce more, larger offspring [36]. But when these better quality individuals are forced to breed in poor environments, their advantage lessens and they produce smaller offspring.

We found no changes in physiological variables in females nesting in high versus low risk sites when the habitat selection decision was controlled. But given free settling decisions [20], the lower condition and physiological stress response of females in high risk sites was likely due, in part, to individual quality gradients with distance from predator nests. Poor quality individuals either showed lower ability to optimize territory location relative to a predator, or poor quality birds that arrived late to breeding sites [47] were more likely to select high risk sites near predators due to density-dependent effects, such as territory defence and conspecific aggression. However, in our previous studies [20], [36] nest-boxes were available in excess and occupation rates suggest selecting other territory locations was possible. Poorer quality individuals showed increased stress responses therefore in the correlative study, but in the current study with quality controlled no significant induction of stress proteins was detected.

The exact extent which an aspect of individual quality accounts for the effect of predation risk is difficult to estimate. But our findings provide field support for laboratory work on *Drosophila* that found stress protein responses are explained by measures related to individual quality. Individuals of lower quality in terms of being inbred [48] or individuals that were exposed to environmental stressors or unfavourable conditions during growth [49], [50] were shown to increase stress protein induction. Low quality individuals generally show higher stress protein induction, and our result with breeding flycatchers support this notion.

Second, our results stress the evolutionary importance of the ability of individuals to gather information regarding the environment prior to making habitat selection decisions. For breeding birds the importance of nest site location decisions has long been appreciated due to the destructive nature of nest predation [13], [14], [51]. But information gathering on adult predation risk prior to territory location decisions also impacts these decisions. Importantly, as for nest site location decisions, even small adjustments in territory location, for example a mere 150 m relative to a breeding predator may have crucial consequences. We forced flycatchers to breed within 50 m of a hawk nest; under normal choice, only 20% of nest boxes were occupied by flycatchers at this distance. In contrast, 65% of nest boxes placed just over 200 m from a sparrowhawk nest were occupied [36].

Our field experiment used ecological realistic manipulations to decrease the quality of habitat in which individuals bred. This study adds to evidence that it is adaptive for prey to cue on the nests of predators when making territory location decisions. This study further suggests that in natural landscapes the decisions of prey will have consequences not only to community structure and diversity, but also to the spatial structure of the quality of individuals. In the case of breeding predators in the landscape, gradient of increasing quality individuals should exist within certain distances of predator nests.

Controlling for individual quality remains a tricky aspect in field studies. We suggest that using proxies of individual quality in analyses may not always adequately account for this variation. While variation in individual quality in the landscape is interesting in its own right, novel field techniques are required that alter decisions made by individuals, to properly measure the effects of aspects of habitat quality.
